# Supplementary material for: Personalized magnetic tentacles for targeted photothermal cancer therapy in peripheral lungs
Source: Commun Eng. 2023 Jul 27;2:50. doi: 10.1038/s44172-023-00098-9 (PMC10955978; doi:10.1038/s44172-023-00098-9)
Supplement: Supplementary file 2 — Supplementary Information [file 44172_2023_98_MOESM2_ESM.pdf]

Title: Magnetic personalized tentacles for targeted photothermal cancer therapy in peripheral lungs.

Authors:
Giovanni Pittiglio[1,2]*†, James H. Chandler[2]†*, Tomas da Veiga[2], Zaneta Koszowska[2], Michael Brockdorff[2], Peter Lloyd[2], Katie L. Barry[3], Russell A. Harris[4], James McLaughlan[5,6], Cecilia Pompili[6,7], Pietro Valdastri[2]

Affiliations:
[1]Department of Cardiovascular Surgery, Boston Children's Hospital, Harvard Medical School, Boston, MA, USA.
[2]STORM Lab, Institute of Autonomous Systems and Sensing (IRASS), School of Electronic and Electrical Engineering, University of Leeds, Leeds, UK.
[3]Leeds General Infirmary – Leeds Teaching Hospital, University of Leeds, Leeds, UK.
[4]Future Manufacturing Processes research group, School of Mechanical Engineering, University of Leeds, Leeds, UK.
[5]Ultrasound Group, Institute of Autonomous Systems and Sensing (IRASS), School of Electronic and Electrical Engineering, University of Leeds, Leeds, UK.
[6]Leeds Institute of Medical Research (LIMR), University of Leeds, Leeds, UK and
[7]Azienda Ospedaliera Universitaria Integrata, University of Verona, Verona, Italy.

*Corresponding authors. Email: giovanni.pittiglio@childrens.harvard.edu, j.h.chandler@leeds.ac.uk.
†These authors contributed equally to this work.

Abstract: Lung cancer remains one of the most life-threatening diseases and is currently managed through invasive approaches such as surgery, chemo- or radio-therapy. In this work, we introduce a novel method for targeted delivery of a therapeutic laser for treatment of tumors in peripheral areas of the lungs. The approach uses a 2.4 mm diameter, ultra-soft, patient-specific magnetic catheter delivered from the end of a standard bronchoscope to reach the periphery of the lungs. Integrated shape sensing facilitates supervised autonomous full-shape control for precise navigation into the sub-segmental bronchi, and an embedded laser fiber allows for treatment via localized energy delivery. We report complete navigation of eight primary lumina in the bronchi of an anatomically accurate phantom (developed from Computed Tomography (CT) data) and successful laser delivery for photothermal ablation. We further evaluate the approach in three diverse branches of excised cadaveric lungs, showing a mean improvement in navigation depth of 37% with less tissue displacement, when compared to a standard semi-rigid catheter, and navigation depth repeatability across all tests of < 1mm.
One-Sentence Summary: Minimally invasive navigation and targeted therapy enabled with personalized design of magnetic tentacles.

SUPPLEMENTARY MATERIALS

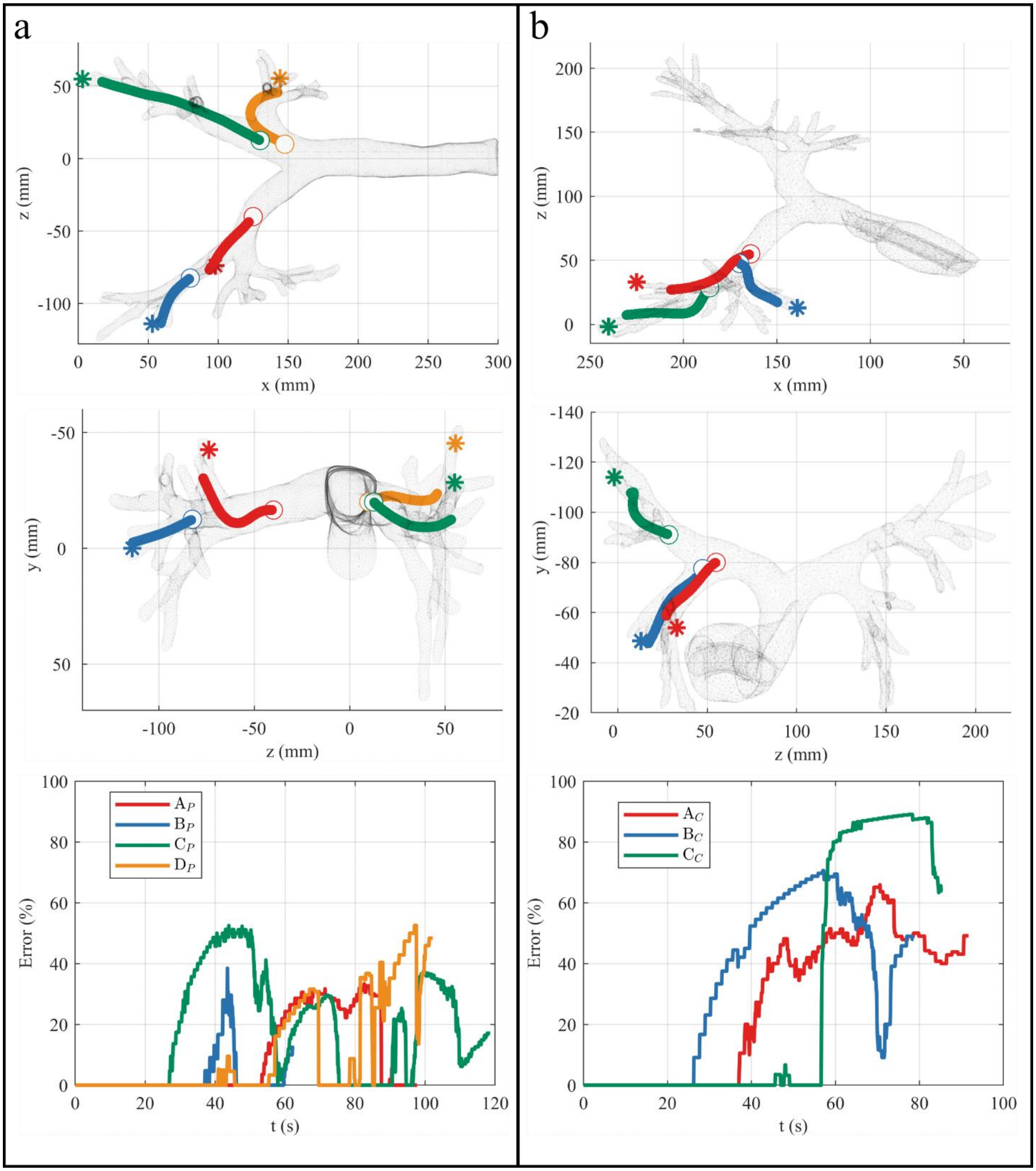

Fig. S1: Localization Results. a Pose and shape sensing overlayed on the frontal, bottom view of the 3D map of the phantom anatomy and percentage of tentacle outside the lumen for 4 sample navigations ((**a**) - (**d**) in Figure 2 represented as red, blue, green and yellow lines respectively). **b** Pose and shape sensing overlayed on the frontal, bottom view of the 3D map of the cadaveric bronchi anatomy and percentage of tentacle outside the lumen.

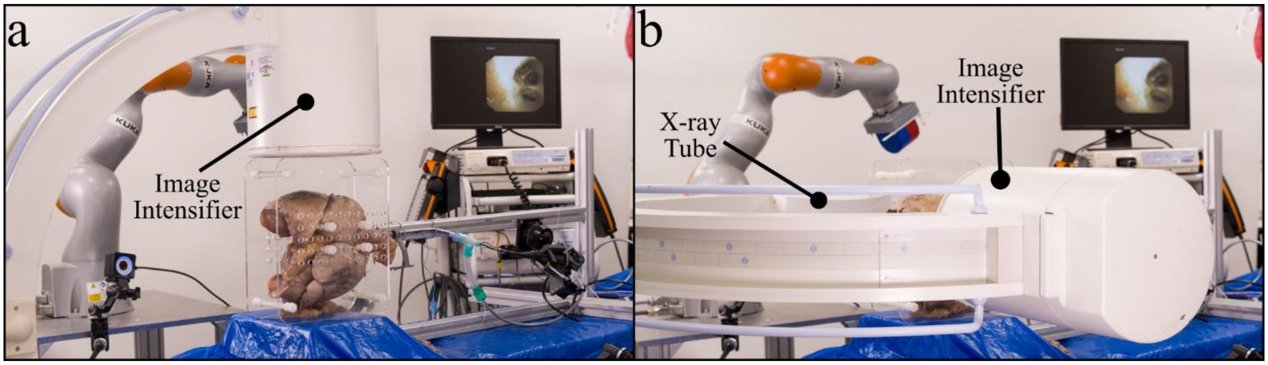

Fig. S2: Fluoroscopy imaging setup. a Image intensifier in Lateral view position. **b** Image intensifier in posterior-anterior view position.

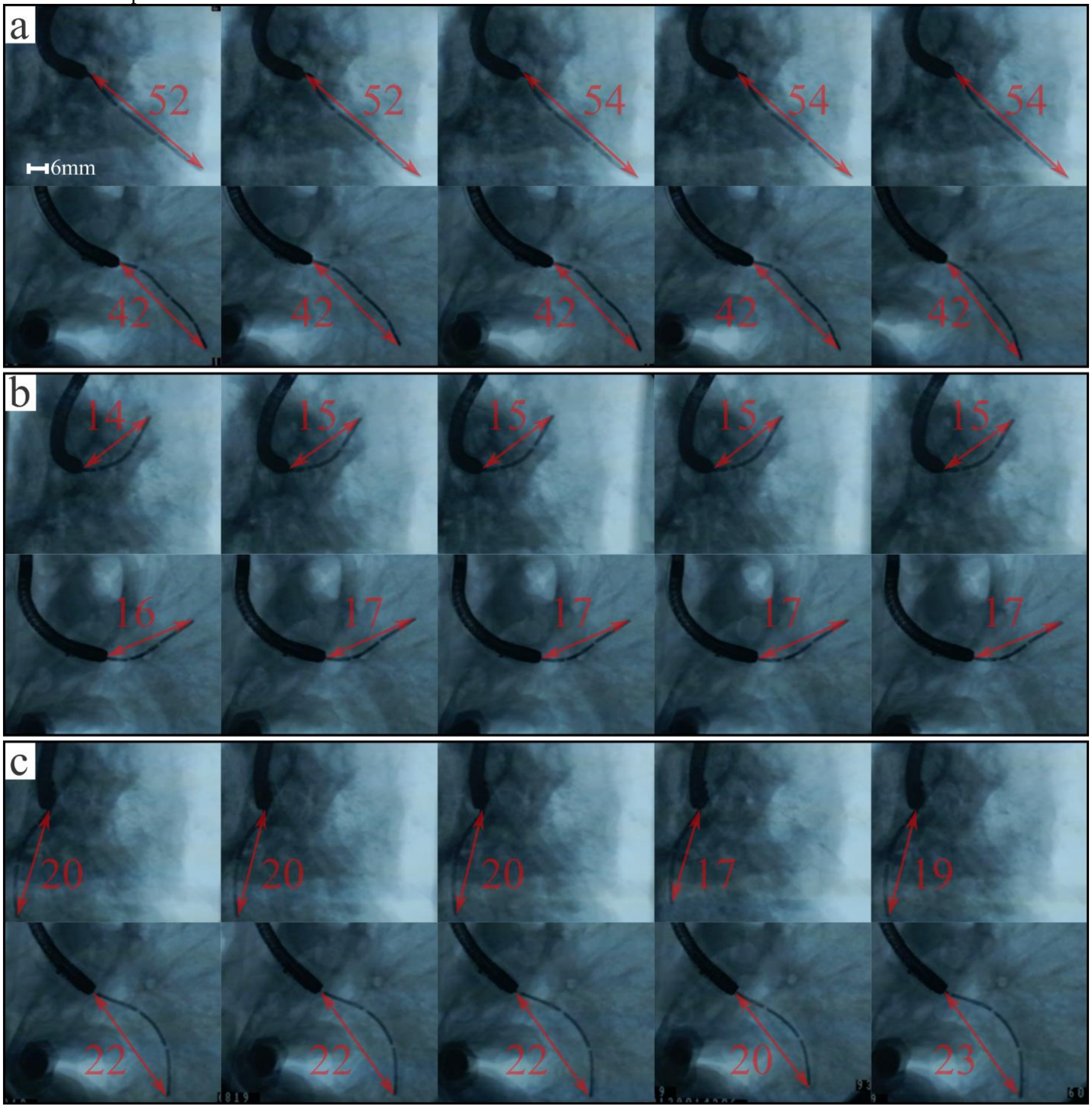

Fig S3: Fluoroscopic images of five repetitions for each navigation in the cadaveric model. Repetitions shown for experiments reported in Fig. 4, including measurement of the penetration depth in millimeters in lateral and posterior-anterior view (see Fig. S2) for three navigation scenarios. **a** Scenario A; **b** Scenario B; **c** Scenario C.

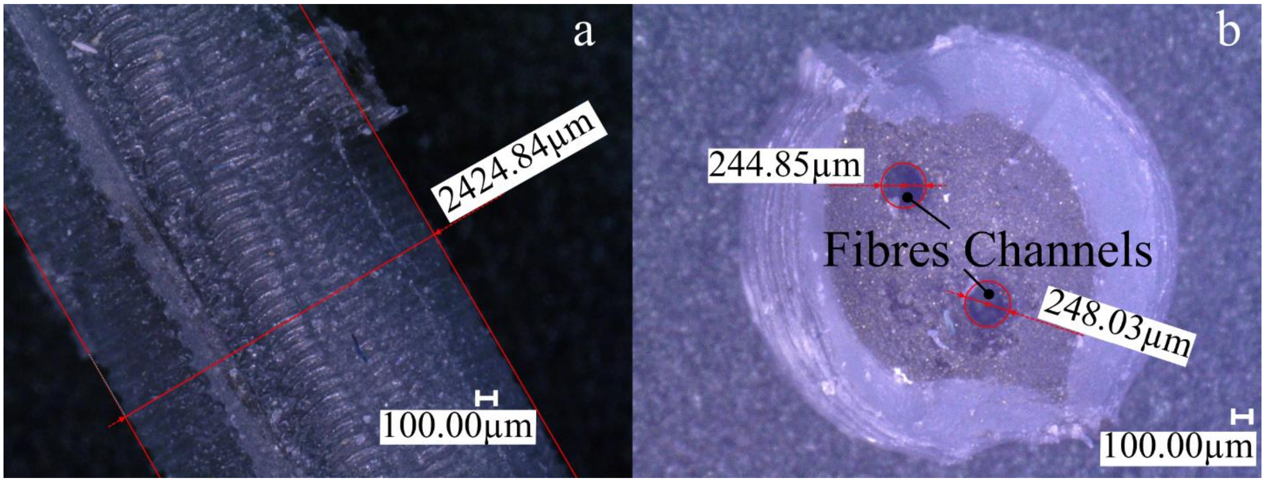

Fig. S4: Analysis of the properties of the fabricated tentacle under optical microscope. Images and optical measurement of the (**a**) lateral view and (**b**) the cross-sectional view of the magnetic tentacle.

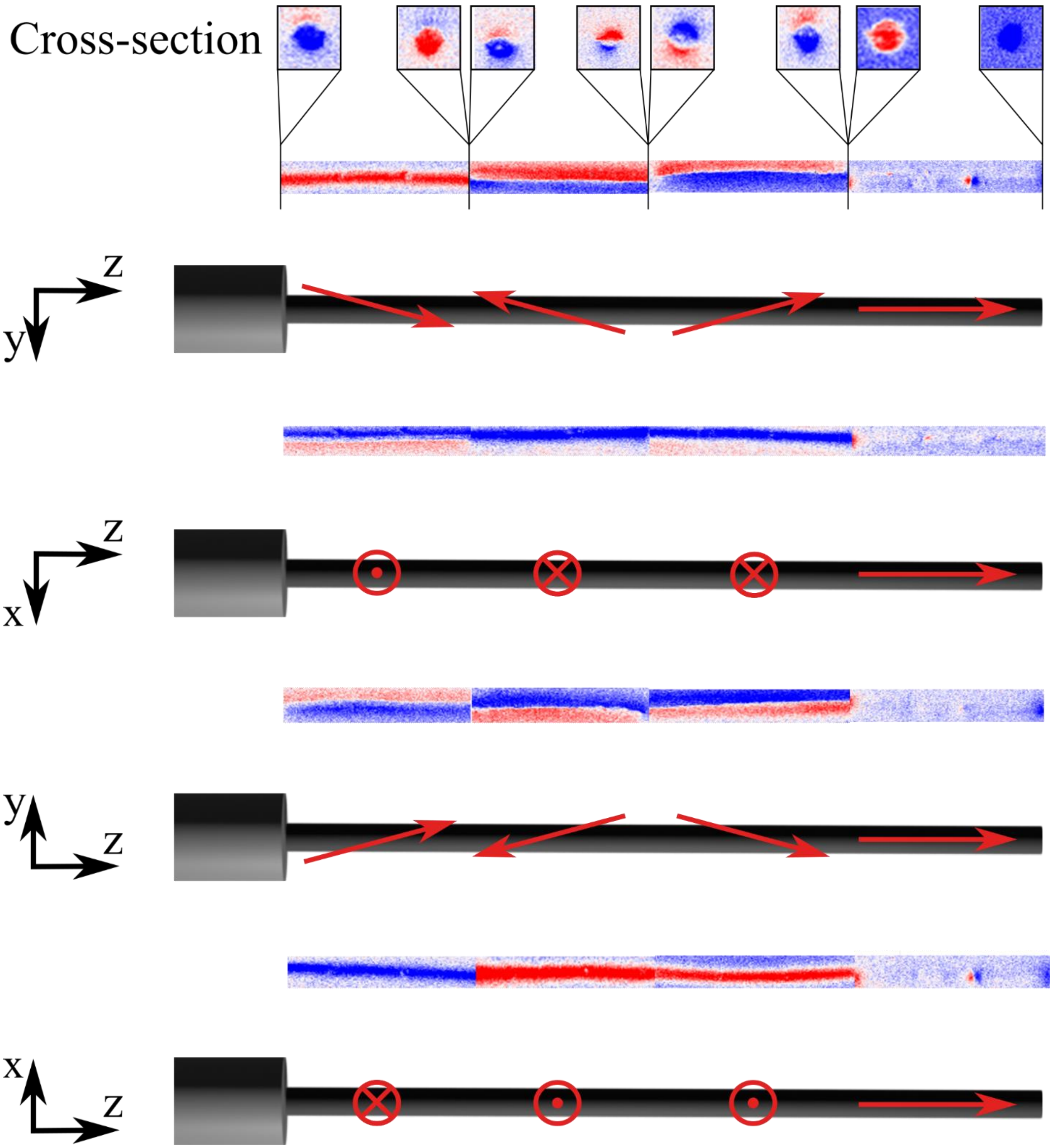

Fig. S5. Analysis of the magnetic properties with Magview. The direction of the arrows follows the pattern of magnetization; it is representative of only the direction not intensity on each plane.
