# Supplementary material for: Personalized magnetic tentacles for targeted photothermal cancer therapy in peripheral lungs
Source: Commun Eng. 2023 Jul 27;2:50. doi: 10.1038/s44172-023-00098-9 (PMC10955978; doi:10.1038/s44172-023-00098-9)
Supplement: Supplementary file 3 — Description of Additional Supplementary Files [file 44172_2023_98_MOESM3_ESM.pdf]

Description of Additional Supplementary Files

File name: Supplementary Movie S1

Description: Phantom: navigation and localization

File name: Supplementary Movie S2

Description: Phantom: detailed navigation.

File name: Supplementary Movie S3

Description: Photothermal energy delivery.

File name: Supplementary Movie S4

Description: Cadaver model: navigation and localization
